# Deciphering Glioblastoma: Fundamental and Novel Insights into the Biology and Therapeutic Strategies of Gliomas

**DOI:** 10.3390/cimb46030153

**Published:** 2024-03-13

**Authors:** Razvan Onciul, Felix-Mircea Brehar, Corneliu Toader, Razvan-Adrian Covache-Busuioc, Luca-Andrei Glavan, Bogdan-Gabriel Bratu, Horia Petre Costin, David-Ioan Dumitrascu, Matei Serban, Alexandru Vlad Ciurea

**Affiliations:** 1 Department of Neurosurgery, “Carol Davila” University of Medicine and Pharmacy, 020021 Bucharest, Romania; razvan.onciul@drd.umfcd.ro (R.O.); corneliu.toader@umfcd.ro (C.T.); razvan-adrian.covache-busuioc0720@stud.umfcd.ro (R.-A.C.-B.); luca-andrei.glavan0720@stud.umfcd.ro (L.-A.G.); bogdan.bratu@stud.umfcd.ro (B.-G.B.); horia-petre.costin0720@stud.umfcd.ro (H.P.C.); david-ioan.dumitrascu0720@stud.umfcd.ro (D.-I.D.); matei.serban2021@stud.umfcd.ro (M.S.); prof.avciurea@gmail.com (A.V.C.); 2 Neurosurgery Department, Emergency University Hospital, 050098 Bucharest, Romania; 3 Department of Neurosurgery, Clinical Emergency Hospital “Bagdasar-Arseni”, 041915 Bucharest, Romania; 4 Department of Vascular Neurosurgery, National Institute of Neurology and Neurovascular Diseases, 077160 Bucharest, Romania; 5 Neurosurgery Department, Sanador Clinical Hospital, 010991 Bucharest, Romania

**Keywords:** gliomas, central nervous system tumors, epidemiology, immunological milieu, tumor microenvironment, transcriptomics, epigenetic alterations, proteomics, metabolomics, comparative oncology

## Abstract

Gliomas constitute a diverse and complex array of tumors within the central nervous system (CNS), characterized by a wide range of prognostic outcomes and responses to therapeutic interventions. This literature review endeavors to conduct a thorough investigation of gliomas, with a particular emphasis on glioblastoma (GBM), beginning with their classification and epidemiological characteristics, evaluating their relative importance within the CNS tumor spectrum. We examine the immunological context of gliomas, unveiling the intricate immune environment and its ramifications for disease progression and therapeutic strategies. Moreover, we accentuate critical developments in understanding tumor behavior, focusing on recent research breakthroughs in treatment responses and the elucidation of cellular signaling pathways. Analyzing the most novel transcriptomic studies, we investigate the variations in gene expression patterns in glioma cells, assessing the prognostic and therapeutic implications of these genetic alterations. Furthermore, the role of epigenetic modifications in the pathogenesis of gliomas is underscored, suggesting that such changes are fundamental to tumor evolution and possible therapeutic advancements. In the end, this comparative oncological analysis situates GBM within the wider context of neoplasms, delineating both distinct and shared characteristics with other types of tumors.

## 1. Introduction

Gliomas are identified as primary CNS tumors thought to originate from neuroglial stem or progenitor cells. These neoplasms have their malignancy graded from 1 to 4 by the World Health Organization (WHO), grades which suggest a range from benign to highly malignant forms [1]. Currently, astrocytoma (AS) *IDH*-mutant is ranked as grade 2, 3, or 4, oligodendroglioma (ODG) is ranked as grade 2 or 3, and GBM, the malignancy with the worst prognostic compared to the latter, as grade 4 [1].

It is well-documented that the majority of low-grade gliomas (LGGs) will transform into high-grade malignant forms over time [2]. The progression of these tumors is conceptualized as a series of developmental stages, which include the initial transformation from the progenitor cell, the acquisition of the capability to invade surrounding tissues, the stimulation of cellular proliferation, the disruption of normal cell cycle regulation, the enhancement of angiogenesis, and subsequent clonal evolution leading to further heterogeneity within the tumor [3].

Gliomas predominantly appear within the cerebral lobes, according to a study performed on 331 adults with a glioma diagnosis: frontal (40%), temporal (29%), parietal (14%), and occipital (3%) [4]. However, a minority may develop in the brain stem, cerebellum, or spinal cord [4]. Epidemiological data indicate a higher incidence of malignant brain tumors in males, whereas females more frequently develop meningiomas and other nonmalignant neoplasms. The median age for diagnosis across all brain and CNS tumors is 59 years [5,6].

Gliomas constitute 24% of adult brain tumors and are considered to be the second-most prevalent CNS neoplasm in this demographic. Survival rates are histology-dependent, with pilocytic AS patients experiencing 10-year survival rates of over 90%, in stark contrast to GBM, where only 5% of patients reach the 5-year survival mark [7].

The occurrence of high-grade gliomas (HGG) in the brainstem is more frequent among females and the non-Hispanic demographic [8]. A significant majority (69.8%) of these neoplasms are identified through radiographic means. Nevertheless, the mortality risk associated with these tumors is increased in Black individuals and those of other races compared to White individuals. Survival rates do not demonstrate a significant variation between sexes [9]. LGGs are categorized as grade 1 or grade 2 neoplasms under the WHO Classification of Tumors of the CNS, a classification that is predicated on their benign characteristics and indolent growth patterns, as evident in radiographic studies. Collectively, these neoplasms constitute the predominant category of primary CNS tumors in the pediatric and adolescent demographic, accounting for approximately 30% of such cases. Within this category, Pilocytic Astrocytomas (PAs; WHO grade 1) are particularly prevalent, comprising around 20% of brain tumors in individuals below the age of 20 years [10,11,12].

Within the context of the overall cancer statistics in the United States, primary brain tumors comprise 1.4% of all cancer cases and account for 2.4% of cancer-related mortality. Annually, there are approximately 20,500 new cases diagnosed and 12,500 fatalities attributed to primary malignant brain tumors [13].

The need for extensive exploration and research in the field comes from the fact that GBM represents the majority of glioma cases, accounting for 57.3% of such diagnoses. The five-year survival rate for patients diagnosed with GBM remains markedly low, standing at a mere 6.8% [14,15].

The recent advancements in the understanding of glioma biomarkers and subtypes have underscored a series of clinical conundrums. One primary challenge lies in the reinterpretation of past study outcomes and retrospective datasets in light of novel classification systems, which is essential for refining prognostic evaluations and therapeutic guidance for patients. Additionally, these new classifications necessitate a reconsideration of the structure and patient stratification methods in upcoming clinical trials [14].

Magnetic resonance imaging (MRI) demonstrates a superior sensitivity in the identification of gliomas; however, the distinction of gliomas from other cerebral pathologies and the precise assessment of their malignancy grade present significant challenges, especially in instances of recurrent gliomas subsequent to earlier therapeutic procedures [16].

## 2. Genetic Landscape of Gliomas

Gliomas have been implicated in association with uncommon genetic disorders that involve germline mutations. Notably, Li-Fraumeni syndrome, linked with mutations in the *TP53* gene, and neurofibromatosis types 1 and 2, underscore the genetic predisposition towards these neoplasms [17].

ASs frequently exhibit mutations in several genes, including *IDH1*, *IDH2*, *ATRX*, *TP53*, and *CDKN2A/B*. ODGs, characterized by *IDH* mutations and a distinctive 1p/19q-codeletion, harbor mutations in *IDH1*, *IDH2*, *TERT* promoter, *CIC*, *FUBP1*, and *NOTCH1*, among others. GBMs, generally *IDH*-wildtype, are identified by mutations in the *TERT* promoter, chromosomal aberrations in chromosomes 7 and 10, and *EGFR* mutations [1].

**Figure 1:**
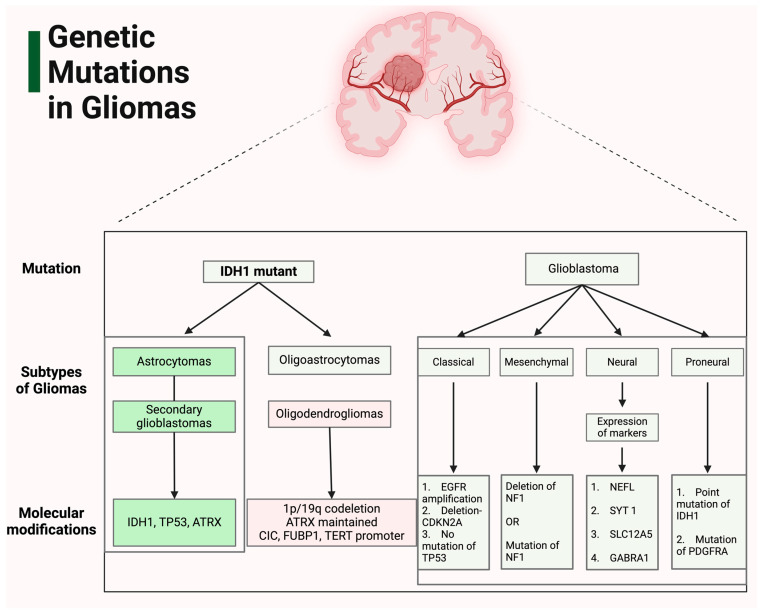
Primary genetic modifications of diffuse gliomas and the genetic alterations of each type of glioblastoma.

Pediatric-type diffuse LGGs, specifically diffuse ASs with *MYB* or *MYBL1* alterations, involve mutations primarily in the *MYB* and *MYBL1* genes. Angiocentric Glioma is similarly associated with *MYB* mutations. Polymorphous Low-Grade Neuroepithelial Tumor of the Young presents mutations in the *BRAF* and *FGFR* gene families. Moreover, diffuse LGGs, showing alterations in the MAPK pathway, have mutations in *FGFR1* and *BRAF* (Figure 1). Pediatric-type diffuse HGGs, particularly Diffuse Midline Gliomas with *H3 K27* alterations, display mutations in *H3 K27*, *TP53*, *ACVR1*, *PDGFRA*, *EGFR*, and *EZHIP*. Conversely, Diffuse Hemispheric Glioma, a *H3 G34*-mutant, exhibits mutations in *H3 G34*, *TP53*, and *ATRX*. Diffuse Pediatric-Type HGGs, both *H3*-wildtype and *IDH*-wildtype, are associated with mutations in *PDGFRA*, *MYCN*, and *EGFR* (methylome). Infant-Type Hemispheric Glioma predominantly shows mutations in the *NTRK* family, *ALK*, *ROS*, and *MET* genes. Among circumscribed astrocytic gliomas, Pilocytic AS is characterized by mutations in *KIAA1549-BRAF*, *BRAF*, and *NF1*, while High-Grade Astrocytoma with Piloid Features exhibits alterations in *BRAF*, *NF1*, *ATRX*, and *CDKN2A/B* (methylome). Pleomorphic Xanthoastrocytoma is distinguished by mutations in *BRAF* and *CDKN2A/B*. Lastly, mutations in *TSC1*, *TSC2*, and *PRKCA* define Subependymal Giant Cell AS and Chordoid Glioma, respectively [1].

The acceleration of biomedical progress has been considerably influenced by the discovery of neomorphic enzymatic activity in the mutated isocitrate dehydrogenase 1 (IDH1), which generates elevated levels of (D)-2-hydroxyglutarate. This oncometabolite is implicated in the initiation and progression of gliomas via epigenetic and metabolic reprogramming. As a result, novel inhibitors targeting the mutant *IDH1* enzyme have been synthesized for therapeutic purposes [18].

Moreover, the use of immunohistochemistry (IHC) for *IDH1*-*R132H*, *ATRX*, and p53 has been employed as a surrogate for genetic status, indicating correlations between histological observations, IHC results, and genetic profiles: (1) IHC for *ATRX* and p53 should augment morphological diagnosis, (2) consideration of *IDH* mutations beyond the common *IDH1 R132H* variant is necessary, and (3) currently, there are no comprehensive substitute assays to fully ascertain the molecular characteristics of GBM as per the 2021 WHO classification [1,19].

In GBMs, *TERT* promoter mutations are prevalent (83%), whereas they are rare in *IDH1*-mutant infiltrating ASs. Given that *IDH*-mutated and *IDH* wildtype gliomas display distinct mutation profiles and clinical trajectories, they are considered to develop through separate oncogenic mechanisms, thus representing distinct entities despite their histopathological similarities [20]. A study by Vriend and Klonisch presents evidence indicating that contributions from genes in the ubiquitin proteasome system (UPS) to the Notch and Hippo pathway signatures are interconnected with stem cell pathways and possess the capability to differentiate GBM from AS and ODG [21]. Their analysis revealed that *AURKA* and *TPX2* (two cell-cycle genes encoding for E3 ligases, as well as the cell-cycle gene responsible for encoding the E3 adaptor CDC20) are upregulated in GBM. Further, genes associated with E3 ligase adaptors, exhibiting differential expression, demonstrated a significant overrepresentation in the Hippo pathway, contributing to the distinction of classic, mesenchymal, and proneural subtypes of GBM [21].

Another comparative study wanted to trace the differences of two very similar gliomas, ODG and AS *IDH*-mutant grade 2. Both of the glioma types showed chromosomal instability, with AS having more total copy number alterations than ODG. ODG specifically exhibited chromosome 4 loss, with the chromosome 7 gain/chromosome 4 loss subtype correlating with a poorer survival and progression-free interval. ODG had a higher subclonal genome fraction and tumor purity, while AS had a greater aneuploidy score. Additionally, AS showed inflamed immune cell infiltration and the potential for immune checkpoint inhibitor response, contrasting with the more homogenous and less aggressive nature of ODG [22] (Table 1).

Recent studies have highlighted *REST* as an oncogenic gene and marker of a poor prognosis in glioma, with its high expression potentially influencing the tumor microenviroment (TME). *REST* expression in glioma was positively correlated with immune cell infiltration and the expression of immune checkpoints, including PD1/PD-L1 and CTLA-4. Furthermore, the analysis identified histone deacetylase 1 (HDAC1) as a potential *REST*-related gene in the context of glioma. An enrichment analysis of *REST* emphasized the significance of chromatin organization and histone modification, suggesting a possible involvement of the Hedgehog-Gli pathway in *REST*’s role in glioma pathogenesis [23,24].

**Table 1:** Important genetic alterations in GBM.

Gene	Physiologic Effect	Mutation	Pathogenicity	Reference
*IDH1*	- Catalyses the oxidative decarboxylation of isocitrate to α-KG and CO_2_, - Converts NAD(P)+ into NAD(P)H.	*IDH1 R132H* mutation is the most common mutation in gliomas	- Derailing of cell metabolism and aiding tumorigenesis. - Elevated 2-HG levels silence tumor suppressors and activate oncogenes. - Inhibiting differentiation of neural cells, promoting a stem-like state and tumor growth. - Metabolic and epigenetic shifts enhance genomic instability. - Reconfiguring the tumor microenvironment to diminish immune responses and bolster tumor viability. - *IDH1* mutations are linked to improved outcomes.	[25,26,27]
*EGFR*	- Tissue development - Cell proliferation - Cellular differentiation - Cellular migration - Celullar survival - Angiogenesis	The *EGFRvIII* mutation and *EGFR* gene amplification are the most prevalent alterations in gliomas	- Common in gliomas, especially glioblastoma, leading to aggressive tumor behavior. - *EGFRvIII* mutation causes continuous activation, driving tumor growth and treatment resistance. - Mutated *EGFR* promotes tumor cell proliferation and survival. - *EGFR* alterations make gliomas resistant to conventional treatments.	[28,29,30,31]
*CDKN2A*	- Controlling cell proliferation - Preventing tumorigenesis—Inducing senescence in response to damage - Maintaining genomic stability - Stem cell regulation	- Point Mutations - Changes in the DNA sequence of *CDKN2A* that impair the function of the *p16^INK4A* protein and its ability to inhibit cell cycle progression - Epigenetic Silencing: methylation of the *CDKN2A* promoter region, leading to decreased expression of *p16^INK4A* and *p14^ARF*, and promoting unchecked cell division and tumor development	- *CDKN2A* mutations lead to *p16^INK4A* loss while undermining CDK inhibition and cell cycle control. - *CDKN2A* mutations determine disrupted cell cycle checkpoints which are pivotal in glioma onset and advancement. - *CDKN2A* mutations underscore the importance of cell cycle governance in glioma prevention and malignancy. - *CDKN2A* mutation status could predict glioma outcomes and therapeutic responses.	[32,33,34]
*TP53* (tumor protein 53)	- Progression of the cellular cycle - Regulates cellular aging	Key hotspots for missense mutations include codons 175, 248, and 273	- Mutations disrupt the DNA damage response. - Compromised *p53* function prevents cell cycle arrest in response to DNA damage. - Altered *p53* fails to trigger apoptosis in abnormal cells and determines a higher survival and proliferation rate of malignant cells. - *TP53* mutations compromise cellular senescence mechanisms. - Mutant *p53* aids in angiogenesis and immune evasion while supporting tumor progression and dissemination. - *TP53* mutation impacts vary, significantly affecting lower-grade gliomas’ evolution into more malignant states. - *TP53* mutations in gliomas frequently lead to resistance against conventional treatments.	[35,36,37,38,39,40,41]
*ATRX*	- Chromatin Remodeling - DNA Repair - Telomere Maintenance - Gene Regulation - Developmental Processes - Neuronal Function	Loss of *ATRX* function can result from various genetic alterations, including mutations, deletions, or gene fusions. This loss is associated with a constellation of molecular changes, notably the *ALT* phenotype, amplification of *PDGFRA*, and mutations in *TP53*	- *ATRX* dysfunction triggers alternative lengthening of telomeres, which determine limitless division of cancer cells. - *ATRX* mutations interfere with chromatin remodeling while altering gene expression, which favors oncogenesis. - *ATRX* loss undermines DNA repair and chromosome segregation, escalating genetic variations conducive to tumor advancement. - Mutations in *ATRX* correlate with particular glioma subtypes, typically signifying an earlier age at diagnosis and unique molecular traits.	[42,43,44,45]
*NF1*	- Cell Growth and Proliferation Regulation Neurofibromin acts as a GTPase-activating protein for RAS, helping to convert active RAS-GTP into its inactive form, RAS-GDP - Cell Cycle Control by regulating RAS activity - Neuronal Development and Function	A predisposing germline mutation in the *NF1* gene often progresses to homozygosity, with the somatic mutation burden in *NF1*-associated gliomas being modulated by both age and tumor grade. High-grade tumors exhibit genetic modifications in *TP53* and *CDKN2A*, alongside prevalent mutations in *ATRX* that are associated with the *ALT* phenotype. Furthermore, these tumors demonstrate an enrichment of genetic alterations affecting transcription/chromatin regulation and the PI3K pathways	- *NF1* mutations diminish neurofibromin function, which leads to sustained RAS signaling. This constant activation fosters cell proliferation, survival, and differentiation. - *NF1* mutations affect neural stem and progenitor cell fate, potentially causing atypical cellular differentiation. - *NF1* functional loss reshapes the tumor microenvironment, enhancing conditions beneficial for tumor growth and survival such as increased angiogenesis for nutrient and oxygen supply. - *NF1* mutations potentially exacerbate genomic instability by compromising DNA repair or fostering a mutation-friendly environment, leading to further mutations that propel glioma progression. - *NF1* mutations grant glioma cells an ability to evade apoptosis.	[46,47,48,49]
*PIK3CA*	Cellular signaling responsible for cellular survival	The localization of *PIK3CA* mutations predominantly to exons 1, 9, and 20 substantiates the notion of mutational hotspots within this gene	- Mutations in *PIK3CA* amplify phosphatidylinositol 3-kinase (PI3K) pathway activity, which boosts cell proliferation, survival, and motility. - The intensified PI3K signaling due to *PIK3CA* mutations disrupts normal cell cycle control, which determines unrestrained cell division. - *PIK3CA* mutations can alter the differentiation pathways of neural stem cells and progenitor cells. - Mutations in *PIK3CA* can modify the tumor microenvironment by enchaning angiogenesis. - *PIK3CA* mutations undermine genomic integrity and facilitate an environment prone to genetic variations. - *PIK3CA* mutations offer glioma cells resistance to apoptosis.	[50,51,52,53]
*PDGFRA*	Transmembrane receptor that is involved in glial proliferation	Two genomic rearrangements, encompassing the starting instance of a gene fusion between the *KDR VEGFR2* and the *PDGFRA* gene, along with six occurrences of *PDGFRAΔ8,9*, an intragenic deletion rearrangement. Notably, the *PDGFRAΔ8,9* variant was prevalent, detected in 40% of GBM cases exhibiting *PDGFRA* amplification	- Almost exclusively in proneural subtype - Mutations in *PDGFRA* lead to enhanced signaling through the *PDGFRα* pathway, which promotes glioma cell proliferation, migration, and angiogenesis.	[54,55,56,57]
*PTEN*	Renowned tumor suppresor gene	Deletions on 10q23. A frequently observed mutational locus resides within exon 5, which is responsible for encoding the phosphatase catalytic core motif. Additionally, recurrent mutations at *CpG* dinucleotides indicate the likelihood of mutations induced by deamination processes	- Hyperactivation of the PI3K/AKT pathway. - Specific to HGG. - Gene mutations correlate with poor prognosis.	[58,59,60]

## 3. The Crucial Role of Glial Cells

Within the CNS, while neurons are primarily responsible for information processing and transmission, glial cells are essential for supporting the neural network’s infrastructure and functionality [61]. Dysfunctions in glial cells can determine a wide spectrum of pathologies, including gliomas. The challenge of treating malignant gliomas stems from two distinct properties of tumor cells: their invasive nature, which precludes total surgical removal even with advanced neurosurgical techniques, and their resistance to standard chemotherapeutic and radiotherapeutic approaches, which allows them to evade complete elimination [62,63]. Furthermore, gliomas initially classified as being low grade frequently undergo malignant transformation within a span from five to ten years, underscoring the urgency for a deeper understanding of their underlying mechanisms to devise effective treatments.

Neural stem cells (NSCs), glial progenitors (including oligodendrocyte progenitor cells), and astrocytes are all potential origins for gliomas. It has been posited that the cells harboring initial mutations may not directly transform; rather, their progeny may undergo transformation, serving as the true cells of origin for these tumors [64]. Research utilizing murine models has demonstrated that, following transduction by oncogenic lentiviral vectors, even highly differentiated brain cells, such as astrocytes and neurons, possess the capacity for dedifferentiation, culminating in the development of GBM [65]. On the other hand, the identification of proliferative cells in the adult brain has led to the hypothesis that GBM may originate from NSCs. This hypothesis has been substantiated through experimental evidence. Specifically, Lee et al. achieved the immortalization of human fetal NSCs using v-myc, followed by the induction of malignant transformation via H-ras to provide oncogenic stimulation. Notably, oligodendrocytes, which originated from the v-myc-expressing progenitor NSCs, did not undergo malignant transformation following oncogenic stimulation by H-ras. Consequently, the authors inferred that NSCs exhibit a higher propensity for neoplastic transformation compared to their differentiated counterparts [66,67].

Extensive research has identified a subset of cells within GBM that exhibit stem cell properties, termed glioma stem cells (GSCs). These cells are implicated in the self-renewal and regeneration of the tumor, contributing to the resilience of the tumor against treatments and its recurrence. Emerging evidence suggests that NSCs located in the subventricular zone (SVZ) are the initial cells of GBM, acquiring the inaugural oncogenic mutations. The involvement of SVZ-NSCs has also been linked to the progression and recurrence of GBM [68]. The SVZ is an ecosystem of NSCs, oligodendrocyte progenitor cells (OPCs), astrocytes, microglia, macrophages, neurons, vasculature, and extracellular matrix (ECM) components. The profound resemblance between NSCs and GSCs lends credence to the theory that SVZ NSCs assume the role of apex cells in the hierarchical organization of gliomas.

The metabolic activity and proliferation rate of tumor cells are typically elevated compared to their healthy counterparts, resulting in a markedly increased demand for iron. This observation underscores the critical role of iron in the development and progression of tumors, and also in GBM [69]. The expression levels of iron-regulated genes (for example, *TfR1* and *TfR2*) are regulated in a different manner between neoplastic brain tissues and normal human brain tissue, with either upregulation or downregulation being observed [70]. Specifically, *TfR2* expression is markedly elevated in GBM cell lines, contributing to enhanced cellular proliferation, with elevated levels of *TfR2* being correlated with an increased sensitivity to temozolomide [71,72,73]. Moreover, STEAP3 plays a pivotal role in iron homeostasis through the reduction of Fe^3+^ to Fe^2+^ [74]. In GBM cells, there is a notable upregulation of STEAP3 expression in comparison to that in normal brain tissues, an expression which consequently makes STEAP3 emerge as a potential prognostic marker, with elevated STEAP3 levels being associated with diminished overall survival (OS) rates [75,76]. Other central mechanisms of ferroptosis are represented by the cysteine/glutathione depletion pathway and polyunsaturated fatty acids (PUFAs). For the cysteine/glutathione pathway, L-cystine is transported into cells via the xc− exchanger, which interacts with glutamate through the SLC7A11 and SLC3A2 carrier proteins [77]. A very recent study conducted by Noch et al. discovered that cysteine compounds, notably N-acetylcysteine (NAC), induce cytotoxicity in GBM cells through promoting mitochondrial hydrogen peroxide production, leading to the induction of reductive stress and diminution of mitochondrial oxygen consumption and membrane potential, culminating in the dissolution of mitochondrial cristae [78]. These phenomena are intensified under conditions of glucose deprivation and be mitigated by the administration of mitochondrial electron acceptors and the overexpression of mitochondrial redox enzymes [78]. For PUFAs to initiate ferroptosis, they must first undergo esterification [79]. Primarily phosphatidylethanolamine PUFAs (PE-PUFAs) are involved in ferroptosis-induced lipid oxygenation, because not all PUFAs represent substrates for it [80]. These PE-PUFAs are synthesized by acyl-CoA synthetase long-chain family member 4 (ACSL4) and lysophosphatidylcholine acyltransferase 3 [81,82]. Subsequently, through the catalytic action of 15-lipoxygenases (LOX) and Fe^2+^, these PE-PUFAs are oxidized into lipid peroxides, facilitating the process of ferroptosis [83,84].

Given the persistently low long-term survival rates exceeding two years, experimental treatments are often integrated with standard care protocols or introduced upon tumor recurrence, which is nearly inevitable [85,86].

Gliomas also exhibit infiltration characteristics similar to those of astrocytes. Astrocytes extend projections to blood vessels and contribute to the blood–brain barrier (BBB). Consequently, when astrocytes undergo oncogenic transformation into gliomas, the resulting tumors can exploit the BBB to support their survival and proliferation [87,88].

## 4. Immuno-Landscape of Gliomas: Understanding the Immunological Milieu

The immune system endeavors to combat glioma cells, yet T-cells exhibit insufficient infiltration within tumor growth regions. Additionally, gliomas express Fas ligand (FasL), a protein that induces apoptosis in immune cells upon interaction. It is anticipated that, by curbing the expression of FasL in brain tumors, the immune system may be empowered to mount an effective response to eradicate the tumor [89,90,91].

**Figure 2:**
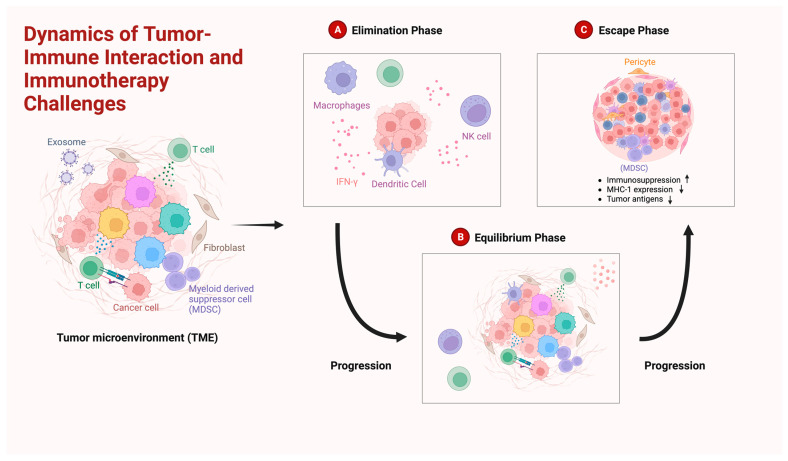
Cancer immunoediting is characterized by three phases: elimination, equilibrium, and escape. In the elimination phase, innate and adaptive immune systems work together to detect and destroy malignantly transformed tumor cells before they become clinically evident. The equilibrium phase represents a balanced state where the immune system keeps tumor cells in check without completely eradicating them, effectively preventing tumor escape. Lastly, the escape phase occurs when tumor growth and proliferation are no longer controlled by the immune system. This leads to a surge in rapidly dividing tumor cells and a shift towards an immunosuppressive environment, disrupting the balance and allowing the tumor to evade immune surveillance.

GBM is very well known for its heterogeneity, meaning that numerous subtypes reside in the tumor [92]. This finding presents a clinical challenge, as the selective elimination of treatment-susceptible clones often leads to the accelerated proliferation of resistant ones. Initiating an immune response targeting a broad range of antigens raises significant concerns due to the potential for antigenic overlap with normal tissue, particularly when using a combination of tumor-associated antigens (TAAs). This dynamic is encapsulated in the ‘Three Es Hypothesis’, an immune editing framework that delineates the continuous interaction between immune cells and tumor cells, encompassing phases of elimination, equilibrium, and escape [93] (Figure 2).

Myeloid-Derived Suppressor Cells (MDSCs) utilize various mechanisms to suppress cytotoxic immune responses in GBM, highlighting their potential as targets in glioma therapies. Studies focusing on microglia and macrophages have revealed their reactions to Glioma-derived Colony-Stimulating Factor-1 (CSF-1) through the use of mouse models, human GBM tumor spheres, and cell lines [94,95]. The role of Arginase 1 positive (Arg1+) exosomes has been investigated in cell culture systems specific to these immune cells [96]. Additionally, the Astrocyte elevated gene-1 (*AEG1*) has been identified as a critical molecule, analyzed through bioinformatic studies of human samples from The Cancer Genome Atlas (TCGA), Genotype-Tissue Expression (GTEx), and the Chinese Glioma Genome Atlas (CGGA), complemented by in vitro cell lines and co-culture methods [97].

Research on MDSCs has been expanded to include analyses of blood samples and tumor tissues derived from patients with GBM, as well as murine models [98]. Macrophage migration inhibitory factor (MIF) has been identified as being influential, with investigations incorporating co-culture assays, GBM patient samples, and syngeneic mouse models [99]. The regulation of Cytotoxic T cells by Programmed cell death protein 1 (PD-1) has been examined through a metadata analysis of glioma samples [100]. Regulatory T cells (Tregs) and T follicular regulatory (Tfr) cells have been studied in relation to PD-1 and Cytotoxic T-Lymphocyte Associated protein 4 (CTLA-4), using human tumor samples, syngeneic mouse models, and tumor cell lines [101]. Tfr cells have been specifically assessed through the analysis of patient-resected glioma samples [102]. The interaction of B lymphocytes with glioma-derived Permeability Factor G (PFG) has been explored in primary cell cultures [103]. The dynamics between Natural Killer (NK) cells, Transforming Growth Factor Beta (TGF-β), and Natural-killer group 2, member D (NKG2D) have been researched using blood samples from glioma patients [104], and the effects of Interferon Gamma (IFN-γ) on NK cells have been explored through human GBM tissue samples [105]. The development of glioma vaccines aims to target tumor-specific antigens similar to those recognized by Chimeric Antigen Receptor T (CAR-T) cells, such as *EGFR* variant III (*EGFR*vIII) and Interleukin-13 Receptor Alpha 2 (IL-13Rα2). Considering the heterogeneity of gliomas and the potential for antigenic variance following treatment, multi-antigen vaccine strategies are being considered, though research to confirm their effectiveness is ongoing [106].

Specifically in glioma, the PD-1/PD-L1 axis is a critical element of immunosuppression within the TME, inhibiting T-cell activation and promoting Treg survival, while glioma cells enhance PD-L1 expression in myeloid cells and Tregs. Additionally, CTLA-4 expression by naive T cells and Tregs serves to limit T-cell proliferation and augment Treg-mediated immunosuppression. Other checkpoints, such as TIM-3 and LAG-3, are also upregulated in GBM, contributing to T-cell exhaustion. Monoclonal antibodies targeting these checkpoints have the potential to revive T-cell functionality and reinstate antitumor immunity [107,108]. The suppression of TIM-3 is bringing new hope in the field of oncology: currently, Sabatolimab represents an innovative immunotherapeutic agent exhibiting immuno-myeloid activity, primarily by targeting the T-cell immunoglobulin domain and mucin domain-3 (TIM-3) on both immune cells and leukemic blasts. This agent is currently under investigation within the ambit of the STIMULUS clinical trial program, specifically for its potential efficacy in the treatment of various myeloid malignancies [109,110]. In the context of glioma, targeting TIM-3 has shown great results. Concomitant administration of an anti-TIM-3 antibody in combination with either Stereotactic Radiosurgery (SRS) or an anti-PD-1 agent demonstrated enhanced survival rates compared to monotherapy with the anti-TIM-3 antibody alone. Notably, the integration of these treatments into a triple therapy regimen culminated in a 100% OS rate (*p* < 0.05), a statistically significant enhancement relative to other treatment arms. This improved survival was concomitant with augmented immune cell infiltration and activity, as well as the establishment of immune memory in long-term survivors, indicating a robust immunotherapeutic response [111]. Ausejo-Mauleon et al. elucidate that TIM-3 is markedly overexpressed in both the tumor cells and immune microenvironment of Diffuse Intrinsic Pontine Glioma (DIPG). Their research indicates that the inhibition of TIM-3 catalyzes a robust immune response, primarily by transforming the DIPG TME into a proinflammatory phenotype. This transformation is mediated through the activation of microglia and CD8+ T cells, thereby fostering an anti-glioma response and facilitating the establishment of durable immunological memory [112]. Ausejo-Mauleon et al.’s results represent a potential therapeutic breakthrough in this domain.

Another different therapeutical approach in oncology is the CCL2-CCR2 axis. CCR2, primarily serving as the receptor for CCL2, exhibits a broad expression profile across a diverse range of cell types, encompassing dendritic cells (DCs), endothelial cells, monocytes, and a variety of cancer cells [113,114,115]. Additionally, findings reveal that CCR2 is also present, albeit at reduced levels, in both neutrophils and lymphocytes [116]. In all these cells, this axis interferes in cellular signaling through the MAPK, JAK/STAT3, or PI3K/AKT pathway [117,118,119]. CCL2 is usually utilized in oncology to determine OS in patients, being, in general, associated with very bad prognostics in gastric cancer, colorectal cancer, pancreatic cancer, and hepatic cancer [120,121,122,123]. Also, this axis is used as a potential avenue of treatment in GBM cell lines. In a study, exosomes derived from irradiated GBM cells substantially facilitated M2 microglial polarization, thereby augmenting the proliferation of these cancer cells. Elevated levels of circ_0012381 in GBM cells, transferred to microglia through exosomes, promote M2 polarization by modulating miR-340-5p and upregulating ARG1 expression. This polarization diminishes phagocytosis and fosters tumor growth via the CCL2/CCR2 axis, with the suppression of these exosomes proving more efficacious in hindering tumor progression than radiotherapy alone in a zebrafish model [124]. Another study demonstrated that targeting MDSCs through CCR2 inhibition can potentiate the effectiveness of checkpoint blockade therapy in GBM. Employing murine models resistant to checkpoint inhibitors revealed that a combination treatment not only diminishes MDSCs, but also amplifies the presence of functional T cells within tumor sites, thereby significantly prolonging OS. These outcomes provide a compelling rationale for the strategic targeting of CCR2-expressing myeloid cells as a means of enhancing immunotherapy efficacy in GBM [125].

## 5. The Importance of Cell Crosstalk in Gliomas

**Figure 3:**
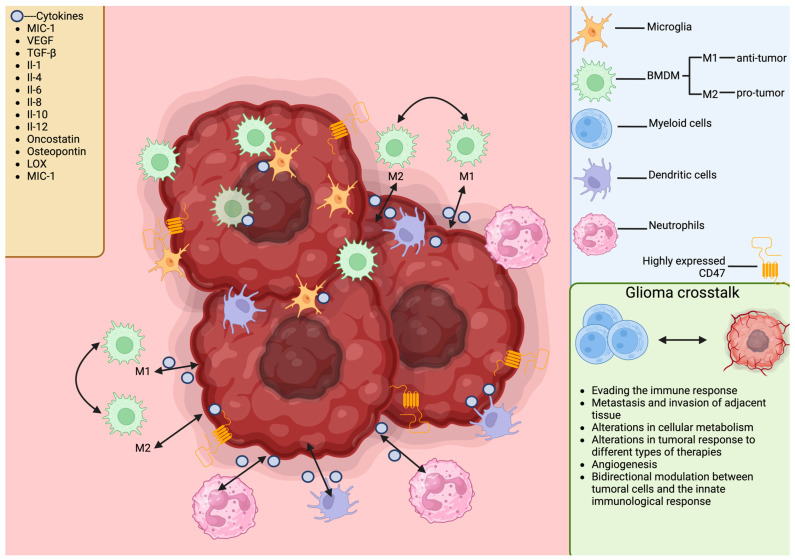
Schematic illustration of the main CNS cells and immune cells that interact in the tumor microenvironment of GBM.

Cellular heterogeneity within a tumor and an immunosuppressive TME act as distinct yet interrelated forces that promote tumor progression and contribute to resistance against therapies. Contemporary research has cast light on the intricate interplay between these intrinsic cellular and extrinsic microenvironmental mechanisms. A prime example of such interactions is the bidirectional communication between cancer stem cells (CSCs) and the infiltrating immune cells within the TME (Figure 3).

GSCs are situated within specialized niches inside the tumor that mirror the critical features of malignant gliomas, such as vascular proliferations and regions of hypoxia/necrosis, and these niches share similarities with the natural microenvironments of physiological brain stem cells. Evidence is accumulating that suggests that complex interactions occur between these niches and glioma cells, with hypoxia being a pivotal factor in the induction of angiogenesis, triggering the upregulation of factors like vascular endothelial growth factor (VEGF), stromal cell-derived factor 1 (SDF1), platelet-derived growth factor (PDGF), and angiopoietins [126].

Additionally, studies have identified a dual role of microglia in high-grade gliomas (HGG). Microglia can exhibit a scavenger-tumoricidal function when activated in an M1 phenotype, whereas M2 phenotype activation is associated with promoting tumor growth and facilitating tumor cell infiltration and migration [127]. The polarization of microglia is influenced by intricate signaling pathways that involve interactions with glioma cells, where extracellular vesicles (EVs) and their microRNA (miRNA) contents appear to be key players. The shift towards a particular microglial phenotype is linked to prognostic outcomes, and the pathological identification of specific microglial states may offer predictive value regarding patient prognosis [128]. Research has demonstrated that human GBM harbors a heterogeneous population of M1/M2 macrophages, with the M1:M2 ratio being linked to an improved prognosis in *IDH1 R132H* wildtype GB [128,129]. Employing automated quantitative immunofluorescence techniques, it was observed that M2-like tumor-associated macrophages (TAMs) are associated with poorer outcomes in HGG, contributing to a microenvironment that supports tumor growth and progression [127,130].

In the context of GBM and other malignancies, EVs facilitate the transport of components between GBM cells and those within the TME, capable of traversing even the blood–brain barrier (BBB), a process facilitated by the presence of Semaphorin3A on their surface. This molecule interacts with neuropilin1 receptors, leading to the disruption of the blood–brain barrier BBB [131,132]. Additionally, gap junctions serve as a crucial communication pathway between astrocytes and GBM cells. In glioma-associated astrocytes, the gap junction protein connexin-43 (CX-43) plays a significant role in enhancing chemotherapy resistance, as well as promoting the proliferation and migration of GBM cells [133,134].

The escalation of c-Jun N-terminal kinase (JNK) pathway signaling in GBM is attributed to the interaction between the receptor Grindelwald (Grnd) and the ligand Eiger (Egr)/TNFα, which is produced by the surrounding non-cancerous brain tissue, according to a study by Portela et al. This study suggests that interactions between GBM cells and adjacent healthy brain tissue may instigate TME expansion, and that such extrinsic signals are instrumental in advancing GBM [135].

Certain brain malignancies establish interactions with neurons, facilitating their advancement. There is emerging evidence indicating a significant function for neuronal cell promoters in the tissue migration of gliomas. Notably, an increased expression of brain-derived neurotrophic factor (BDNF) has been linked to the pathological advancement of gliomas [136]. BDNF, a recognized synaptic modulator, is involved in various neurological functions, including the enhancement of memory and neuronal plasticity. Research by Wang et al. has demonstrated that an interaction between GBM cells and the BDNF receptor, NTRK2, forms a complex that is crucial to the tumor progression process [137]. Paracrine signaling is the neuronal activity that propels tumor progression, in the context of gliomas, via neuroligin-3 and BDNF [138,139,140], as well as through neuron-glioma synapses mediated by AMPA receptors, synapses which are modulated by BDNF [141,142,143]. Signaling through the receptor TrkB to CAMKII, BDNF facilitates the trafficking of AMPA receptors to the glioma cell membrane [144]. This process enhances the amplitude of glutamate-evoked currents within malignant cells, thereby contributing to glioma progression [144].

Lately, estrogens have garnered a lot of attention due to the possibility of contributing to the development of GBM. Estrogens support cell proliferation and tumor growth through the presence of G protein-coupled estrogen receptor (GPER), which has been identified in C6 GBM cells [145], as well as in AS cell lines U251 [146]. Moreover, estrogen has been identified in the TME [147]. In GBM, the functions of two estrogen receptors, ERα and ERβ, diverge significantly [148,149]. ERα-36, a splice variant of estrogen receptor alpha (ERα-66), has been implicated in mediating cell proliferation through both estrogenic and anti-estrogenic signaling pathways across various cancer types. ERα-36 expression is elevated in GBM cells [150,151]. Among ERβ isoforms, ERβ5 emerges as the predominant variant identified in gliomas [152,153]. The expression of ERβ5 is upregulated under hypoxic conditions within the glioma microenvironment, functioning as a self-protective mechanism to curb tumor proliferation. Selective ERβ agonists, such as MF101 and liquiritigenin, together with histone deacetylase inhibitors, suppress glioma cell proliferation and inhibit tumor growth, highlighting their therapeutic efficacy in glioma treatment strategies [154,155,156].

To date, therapeutic strategies targeting specific cellular elements or intracellular metabolic pathways have not yielded improvements in patient survival rates for GBM. GBM has the capacity to co-opt healthy brain cells, manipulating their functions to foster a conducive microenvironment that augments tumor progression. This microenvironment forms an intricate network where malignant cells not only interact among themselves, but also with normal and immune cells, fostering tumor growth, angiogenesis, metastasis, immune evasion, and resistance to therapy. The modes of communication within this network range from direct cell-to-cell contact via adhesion molecules, tunneling nanotubes, and gap junctions to indirect interactions through paracrine signaling, utilizing cytokines, neurotransmitters, and EVs [157,158].

A study by Jeon et al. establishes that the PDGF–NOS–ID4–miR129 regulatory axis stimulates the JAGGED1–NOTCH signaling pathway in glioma-initiating cells (GICs) and endothelial cells [159]. The activation of JAGGED1–NOTCH signaling is implicated in promoting tumor advancement, characterized by an enhanced proliferation of GICs and increased angiogenesis. These observations also offer a therapeutic premise for targeting NOTCH signaling pathways, which are essential for the sustenance of tumor perivascular microenvironments comprising GICs and endothelial cells [159].

Targeting CX43 presents a substantial challenge due to the obscurity of the mechanisms through which CX43 mediates resistance. Notably, CX43 is expressed at high levels in GBM, a condition which correlates strongly with poor prognoses and resistance to chemotherapy. The suppression of CX43 expression via miR-1 has been shown to impede the infiltration and proliferation of glioma cells. Additionally, this downregulation of CX43 expression facilitates the induction of apoptosis in glioma cells, further elucidating the regulatory role of CX43 in these processes [160].

Aquaporins (AQPs) constitute a family of membrane channel proteins, which are responsive to variations in osmotic or hydrostatic pressure, thereby enabling the transcellular movement of water across biological membranes. To date, over ten functional isoforms of human AQPs have been identified, each uniquely expressed in various bodily regions and possessing distinct characteristics and functional roles [161]. AQPs represent potential targets in cancer therapy. For example, a study aimed to find out if tumor motility could be suppressed by inhibiting AQP-1 and subsequent ionic channels. The results showed that natural compounds like xanthurenic acid and caelestine C or semi-synthetic amides decreased glioma infiltration in the 251-MG and U87-MG GBM cellular lines [162]. The functions of AQP1 in regulating cell volume have been suggested to facilitate morphological adaptations in glioma cells, transforming them into elongated spindle shapes. This alteration enables glioma cells to navigate through the constricted extracellular spaces in the brain [163]. Additionally, a separate study identified a strong association between AQP4 expression and the expressions of epidermal growth factor receptor (EGFR), 4-aminobutyrate aminotransferase (ABAT), and platelet-derived growth factor receptor alpha (PDGFRA) in the classification of GBM. These factors have been proposed as potential targets for AQP4-related immunotherapy strategies [164]. Tan et al. documented a negative correlation between the apparent diffusion coefficient and the expression of Aquaporin 4 (AQP4) mRNA in AS [165].

## 6. Transcriptomics of Gliomas

Recent advancements in RNA sequencing technologies and sophisticated data analysis have facilitated the characterization of comprehensive transcriptomic landscapes. These include profiling protein-coding and non-coding gene expressions, discerning alternative splicing events, and detecting fusion genes, thereby advancing disease detection and the understanding of altered phenotypes [166]. Differentially spliced cancer drivers encompass elements of the RAS/MAPK pathway. Specifically, neurofibromin 1, a suppressor of RAS, undergoes alternative splicing, resulting in a less active isoform in over 80% of HGGs. This splicing variant emerges downstream from *REST* upregulation, leading to the activation of the RAS/MAPK pathway and consequently diminishing survival rates in patients with GBM [167].

The formation of neurospheres (NS) is marked by the activation of five transcription factors (TFs) commonly associated with gliomas: *SOX2*, *UBTF*, *NFE2L2*, *TCF3*, and *STAT3*. Concurrently, the transcriptional activity of the TFs *MYC* and *MAX* is diminished in NS [168]. Genes that are upregulated are implicated in processes such as epithelial–mesenchymal transition, cancer stemness, and the invasive and migratory behaviors of glioma cells [168]. Conversely, genes downregulated by *MYC/MAX* are involved in translation, focal adhesion, and apical junctions. Additionally, the study by Vasileva et al. identified three regulators—SPRY4, ERRFI1, and RAB31—that are common feedback elements in EGFR and FGFR signaling across the gliomas analyzed, offering potential targets for developing therapeutic strategies to inhibit glioma infiltration and progression [168].

The long non-coding RNA (lncRNA) CRNDE exhibits pronounced upregulation in glioma tissues and has been implicated in enhancing proliferation, migration, and infiltration processes [169]. CCDC26, another lncRNA, is newly identified and demonstrates increased expression in glioma tissues, exerting its influence by directly targeting miR-203, as demonstrated in both in vitro and in vivo studies [170]. Furthermore, the in vivo downregulation of ATB has been shown to inhibit tumor growth. Additionally, miR-152, an miRNA, is down-regulated by H19 [171], leading to the stimulation of tumor proliferation and infiltration, as observed in vitro and in vivo [172]. Moreover, limited information is available regarding *ADAMTS9-AS2*, a gene whose expression is observed to be downregulated in glioma, correlating with the grade of the glioma. Studies have demonstrated that the overexpression of *ADAMTS9-AS2* results in the inhibition of cellular migration and infiltration processes [173].

Another study also indicated that most transcriptional changes in tumor samples are not dependent on deoxyribonucleic acid (DNA) methylation. Instead, altered histone *H3* trimethylation at lysine 27 (*H3K27me3*) emerged as the primary molecular anomaly in deregulated genes. It is proposed that the presence of a bivalent chromatin signature at *CpG* island promoters in stem cells is predisposed not only to hypermethylation, but to a broader spectrum of transcriptional disturbances in transformed cells. Furthermore, it was observed that the level of gene expression in normal brain cells significantly impacts the mechanism of transcriptional repression utilized in glioma, with genes that are highly expressed in healthy cells being more susceptible to silencing through *H3K27me3* modification rather than DNA methylation. These findings underpin a theoretical framework wherein altered *H3K27me3* dynamics, particularly through the interaction between polycomb protein complexes and the brain-specific transcriptional apparatus, are primarily responsible for the transcriptional deregulation evident in glioma cells [174].

Diffuse lower-grade LGGs are marked by extensive genetic and transcriptional diversity. However, the variability within their DNA methylation profiles, their functional role in tumor biology, their integration with the transcriptome and the TME, and their influence on tumor evolution remain underexplored. The study uncovered parallels between AS-like LGGs and grade 4 *IDH*-wildtype gliomas in terms of the potential exacerbation of treatment resistance progressing along a proneural-to-mesenchymal trajectory. Through the use of gene-signature-based analysis, the influence of the cellular composition of tumors was also delineated, including the role of immune cell bystanders like macrophages [175].

Additionally, methylation of the *MGMT* promoter serves as a predictive marker for the efficacy of alkylating chemotherapy in patients with GBM. Predictive biomarkers for targeted therapies are also gaining prominence, including mutations in *IDH1* and *BRAF* [176].

Gliomagenesis and tumor progression are critically dependent on the dysregulation of TFs reviewed herein. These central regulators govern processes such as glial differentiation, stress response adaptation, cell cycle regulation, and angiogenesis, all contributing to the malignancy and recurrent nature of gliomas. They have become increasingly recognized for their potential as therapeutic targets and as prognostic indicators [177,178].

## 7. Emphasizing Epigenomics in Glioma Research

Epigenetic modifications exert a profound influence on gene expression and, consequently, on the development and behavior of glioma cells. Previous research has extensively documented that changes in DNA methylation represent one of the most well-characterized epigenetic alterations in human pathology. Global DNA methylation patterns in gliomas interact with histone modifications, which may influence TFs, affect global gene expression, and alter chromatin structure [179,180].

Additionally, histone posttranslational modifications (PTMs) are integral in regulating chromatin structure and gene expression, significantly impacting malignant transformation, tumor growth, and progression. Variations in the expressions of the genes responsible for encoding enzymes involved in methylation (such as *G9a*, *SUV39H1*, and *SETDB1*) and acetylation/deacetylation (including *KAT6A*, *SIRT2*, *SIRT7*, and *HDAC4*, *6*, and *9*) are implicated in the pathogenesis of GBM. Moreover, SUMOylation pathway proteins are notably upregulated in GBM cell lines, encompassing E1 (SAE1), E2 (Ubc9) components, and a SUMO-specific protease (SENP1) [181]. It is interesting to note that GSCs, when cultured in immunocompetent hosts, undergo an epigenetic adaptation process, culminating in the secretion of immunosuppressive cytokines [182], additionally exhibiting an upregulation of IRF8, a cytokine typically confined to myeloid cells, given its regulatory role in myeloid lineage control and macrophage differentiation.

Furthermore, miRNAs, small non-coding RNA molecules, are recognized for their critical roles in a variety of biological processes, including cell growth, proliferation, tumor infiltration and metastasis, apoptosis, angiogenesis, and immune responses. Significant strides have been made in researching the miRNA pathways associated with GBM pathogenesis. miRNAs have been identified as potential diagnostic and prognostic biomarkers and are increasingly being considered as therapeutic targets and agents [183,184,185].

**Figure 4:**
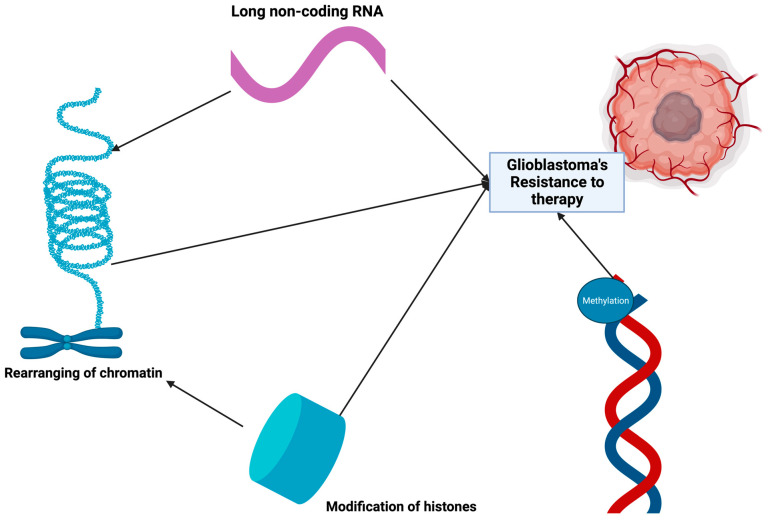
A diagrammatic overview of the relationship between various epigenetic mechanisms and the development of therapeutic resistance in glioblastoma would illustrate that DNA methylation, histone modifications, chromatin remodeling, and long non-coding RNAs are integral in conferring resistance. These epigenetic factors contribute through diverse pathways: they enhance cell proliferation, inhibit apoptosis, foster stem-like characteristics, reduce the efficacy of DNA damage repair, stimulate autophagy, and facilitate the epithelial–mesenchymal transition.

Epigenetic reconfiguration is a defining characteristic of gliomas, playing a pivotal role in their progression. This dysregulation is central to the initiation of gliomas, their evolutionary trajectory, their interaction with immunotherapies, and is also indicative of clinical outcomes (Figure 4) [186,187,188,189].

Kanamori et al. indicated that Notch activation could enhance the aggressive traits of GBMs. The expression levels of Notch 1 correlate with GBM patient survival and Notch 4 expression is linked to GBM [190,191,192,193].

The Notch signaling pathway is characterized by four types of cytoplasmic receptors, specifically the homologous proteins Notch 1–4, which are situated on cells to receive signals. These are complemented by their ligands, encompassing the Delta-like family (DII1–4) and the Jagged family (Jagged 1 and 2), located on signaling cells. The distribution of these receptors is extensive throughout the adult brain. Notch 1 is found in neurons, astrocytes, precursor cells, ependymal cells, and endothelial cells. The expressions of Notch 2 and 3 are primarily in precursor cells. DII1 is present in intermediate neuronal progenitors and neurons, and DII3 in intermediate neuronal progenitors [194,195,196,197,198]

## 8. Proteomics and Metabolomics

Recent technological advancements have catalyzed the discovery of novel molecular mechanisms that drive the altered metabolism characteristic of gliomas [199,200,201].

Proteomics, which encompasses the study of proteins, their structures, functions, interactions, and cellular activities, offers a more detailed understanding of an organism’s biological processes compared to genomics. The complexity of proteomics surpasses that of genomics because protein expression varies with time and environmental factors [201,202].

Gliomas form as a result of cellular mutations that disrupt normal metabolism, leading to uncontrolled cell growth. Specific mutations in genes such as *EGFR*, *PDGFRα*, and *PTEN* activate the protein kinase B (PKB/Akt) pathway, which then stimulates the mTORC-1 pathway, crucial for protein metabolism and thus cell proliferation. Additionally, glioma proliferation is influenced by other mechanisms, including RTK/Ras/ERK signaling and alterations in telomerase activity [203].

The S100 protein family, with 25 members, is characterized by the Ca^2+^ binding EF-hand motif and has been associated with disease progression, diagnosis, and prognosis, particularly in tumors. These proteins have diverse roles both inside and outside the cell, affecting processes like cell proliferation, differentiation, motility, enzyme function, immune response, cytoskeletal dynamics, calcium homeostasis, and angiogenesis [204].

Furthermore, the transcript levels of S100A8/S100A9 are recognized as independent indicators of a poor prognosis in GBM. Elevated pre-operative and three-month post-operative serum levels of S100A8 predict a less favorable prognosis in GBM patients who surpass the median survival rate. Laboratory studies have demonstrated that recombinant S100A8/S100A9 proteins can enhance glioma cell migration and infiltration through integrin signaling up to certain concentration thresholds [205].

The pursuit of personalized medicine continues, with researchers focusing on targeted therapies that are tailored to the unique attributes of each patient’s condition. Metabolic and lipidomic studies are among the most promising avenues for this endeavor, as they provide granular analyses of alterations in the small molecule composition within a biological system or sample [206].

The metabolic profile of cancer cells is shaped by various factors, including elevated glucose uptake, hypoxic conditions, and the presence of infiltrating immune cells, all of which must be taken into account to optimize treatment strategies [207,208].

GBM, along with many other cancers, is characterized by a distinct bioenergetic profile known as the Warburg effect, where cells preferentially utilize aerobic glycolysis for energy [209]. In certain pre-clinical breast cancer models, ketones have been shown to support tumor growth through a reverse Warburg effect and metabolic coupling between stromal and cancer cells [210,211,212]. Therefore, the metabolic pathways underpinning rapid tumor growth are increasingly being recognized as viable targets for cancer therapy. Moreover, the Warburg effect can be used to assess the prognosis and microenvironment of the glioma [213].

The PI3 kinase (PI3K) family is intricately involved in cellular processes and metabolism. Many cancers, including GBM, frequently exhibit activated PI3K signaling due to mutations that activate *PIK3CA* or inactivate *PTEN* [214]. Insights into the roles of PI3K’s regulatory and catalytic subunits in metabolism and oncogenesis have been gained through the use of genetic mouse models and small molecule inhibitors [215,216]. The cascades from receptor tyrosine kinases through PI3K to Akt and mTOR are particularly significant in GBM and serve as potential therapeutic targets. The clinical introduction of small molecule inhibitors targeting these kinases offers a hopeful treatment modality [217].

Additionally, a phenomenon known as glutamine addiction reflects a metabolic adjustment that complements oxidative glycolysis, providing neoplastic cells with nutrients and energy, particularly under hypoxic conditions. Various clinical approaches are being explored to disrupt glutamine metabolic pathways in gliomas [218,219,220].

As mentioned before, the Warburg effect promotes that cancer cells consume glucose at high rates and produce substantial amounts of lactate, even in aerobic conditions [221,222]. GBM exhibits a pronounced dependency on glycolysis within the TME [223,224,225]. Stimulation of the glycolytic pathway in GBM is directly proportional to its progression [226]. Consequently, numerous therapeutic strategies are under development to counteract the Warburg effect. Research has indicated that the silencing of PDIA4 can impede the PI3K/AKT/mTOR pathway-dependent cell proliferation and induce apoptosis, coupled with a reduction in the Warburg effect [227]. Furthermore, Poteet et al. illustrated the potential of methylene blue to reverse the Warburg effect in GBM by facilitating the transfer of electrons from NADH to cytochrome c in mitochondrial complex I, thereby diverting pyruvate into the citric acid cycle [228].

Acetyl-CoA synthetase 2 (ACSS2) facilitates the conversion of cytosolic acetate into acetyl-CoA, serving as a precursor for the de novo biosynthesis of fatty acids and cholesterol, thereby supporting tumoral proliferation [229,230]. Notably, ACSS2 expression is elevated in GBM, as well as in other malignancies, including hepatocellular carcinoma, bladder, and prostate cancers [231]. Additionally, acetyl-CoA plays a crucial role in epigenetic modulation, as it translocates into the nucleus to effectuate histone protein modification through direct acetylation [232]. In GBM, there is a significant upregulation of fatty acid synthesis, in a mechanism that facilitates accelerated tumor proliferation [233,234,235]. The oncogenic EGFR/PI3K/AKT signaling cascade augments fatty acid biosynthesis through the stimulation of sterol regulatory element-binding protein-1 (SREBP-1) activation.

## 9. Surgical Treatment

Findings from multiple studies have significantly highlighted the criticality of achieving complete resection in the treatment of LGGs. The extent of resection (EOR) not only impacts OS in patients with LGGs, but also plays a role in influencing the rate of malignant transformation and the likelihood of achieving seizure-free status [236]. A retrospective analysis involving 153 glioma patients, comparing outcomes between those who followed a “watch and wait” approach and those who underwent early surgical resection, revealed that patients with LGGs who had early surgery exhibited higher survival rates. This suggests the critical importance of the timing of the resection [237]. In patients with HGG, retrospective analyses have established a significant correlation between the extent of tumor resection and both OS and progression-free survival. Moreover, the observation that incomplete resections lead to more rapid neurological decline further underscores the pivotal role of complete tumor resection in enhancing progression-free survival outcomes [238]. Awake craniotomy for the surgical excision of gliomas in eloquent brain regions has been shown to positively impact the EOR, survival rates, postoperative neurofunctional outcomes, and length of hospital stay (LOS). Given these benefits, awake craniotomy is a preferred surgical approach, when applicable, for resecting gliomas [239]. Local recurrence is a principal factor contributing to the malignant progression of GBM in clinical settings, with the majority of recurrences occurring within a limited margin (2 to 3 cm) surrounding the tumor cavity. To enhance the rate of complete surgical resection (CSR), numerous advanced therapeutic techniques have been developed, such as multimodal MRI, intraoperative MRI (IoMRI), intraoperative ultrasound, and fluorescence-guided surgery [240,241,242]

Despite extensive post-surgical management, the complete eradication of disseminated tumor cells around the tumor cavity remains unachievable under microscopic observation. Intraoperative radiation therapy (IORT) represents a strategic approach involving the application of high-dose radiation directly to the residual tumor bed during surgery. This method minimizes damage to the surrounding healthy brain tissues and is advantageous for eliminating residual tumor cells at the surgical resection margins [243]. IoMRI significantly revolutionizes the approach to glioma treatment. This technology not only addresses the issue of brain shift during surgery, but also aids neurosurgeons in identifying residual tumor tissue, thereby facilitating a higher EOR. In a prospective study of 100 adult patients undergoing glioma surgery with IoMRI and neuronavigation, Leroy found that the median EOR was consistently 100% across different types of gliomas and their locations, with the exception of increased residue in the insular area. The study also noted no variance in the median Karnofsky Performance Status (KPS) between patients with LGG gliomas and HGG at various postoperative follow-up periods. Leroy introduced the concept of “staged volume” surgery, aiming to maximize safety for surgeons and minimize morbidity for patients [244].

## 10. The Tumor Microenvironment in Gliomas: A Focal Point

The TME niche encompasses a diverse array of cellular constituents, including endothelial cells, various neural cells, and resident and infiltrating immune cells, as well as noncellular elements like signaling molecules, exosomes, and ECM components. The glioma TM constitutes a dynamic and heterogenous system involving direct and indirect cellular communication, with factors such as pH and oxygen concentration also playing pivotal roles in its modulation [245,246].

Critical to the functioning of the immune system is intercellular communication among immune cells, facilitated by direct cell contact or through soluble mediators like cytokines and chemokines. Cytokines exhibit a range of activities—autocrine, paracrine, and endocrine—while chemokines additionally influence T cell differentiation and leukocyte extravasation, contributing to tumor progression [247,248]. Tumor cells also utilize various methods such as exosomes, gap junctions, and horizontal DNA transfer to interact within the TME. Recognizing the significance of the TME and the intricate network of communication between tumors and normal cells has led to new therapeutic strategies targeting GBM [249].

Moreover, the dense and highly interconnected ECM presents both direct and indirect challenges to treatment efficacy. Its rigidity can act as a physical barrier, hindering drug delivery to tumor cells and effectively shielding the tumor from therapeutic agents. Additionally, this ECM density can restrict the diffusion of nutrients and oxygen, further complicating treatment strategies [250]. Venkatesh et al. discovered that OPC-like glioma cells express synaptic genes [251]. They identified two types of synapses between glioma cells and neurons: one resembling neuron-OPC synapses mediated by AMPA receptors and another akin to neuron-astrocyte synapses mediated by potassium currents. GluA2, an AMPA receptor component, was found to enhance glioma growth and reduce survival in vivo; thus, neuron-glioma synaptic connections were shown to be crucial for glioma progression, infiltration, and proliferation [200]. TREM2 (triggering receptor expressed on myeloid cells 2), which is normally expressed on the brain’s immune cells, is associated with a poor prognosis in glioma if overexpressed in macrophages and microglia [252].

The involvement of cancer-associated fibroblasts (CAFs) in tumor progression is complex and multifaceted. Analogous to immune cells that initially inhibit malignant growth, CAFs constrain early tumor progression, predominantly through the establishment of gap junctions between activated fibroblasts. However, as the tumor evolves, CAFs become activated by various tumor-secreted factors, subsequently facilitating tumor growth and advancement. This dynamic is characterized by two interrelated pathways in the interaction between cancer and stromal cells: firstly, the “efferent” pathway, where cancer cells elicit a reactive response from the stroma, and secondly, the “afferent” pathway, wherein the altered stromal cells within the TME modulate the responses of cancer cells [253,254,255,256]. Until quite recently, CAFs were thought to be absent in GBM, one key argument being that fibroblasts are not present inside the brain parenchyma. However, a series of studies set out to determine this supposition, which was, in the end, true; CAFs are attracted by GBM stem cells [257,258]. Galbo Jr. et al.’s research delineated a significant correlation between the abundance of CAFs and a higher tumor grading, adverse clinical outcomes, and the activation of ECM remodeling processes. Analyzing the secretome of these CAFs, fibronectin (FN1) emerged as a potent mediator of CAF functions. These findings were derived from comprehensive analyses conducted in both in vitro and in vivo model systems [259].

The initial phase of tumor–immune interaction, known as the elimination phase, involves the immune system’s recognition and eradication of transformed cells. Should these cells circumvent immune detection during the elimination phase, they may proliferate into tumors. As the tumor and its stroma evolve, the mechanisms of immunosuppression become more pronounced. Despite the immune system’s capacity to recognize and destroy tumor cells, the tumor may continue to expand during what is referred to as the equilibrium phase, eventually progressing to the escape phase, where it evades immune surveillance altogether [260]. Molecules released by the tumor sculpt the TME, fostering an immunosuppressive state that undermines effective antitumor immune responses. Within the TME, MDSCs represent significant barriers to cancer immunotherapy, diminishing the efficacy of antitumor actions and conferring increased resistance to immunotherapeutic approaches on tumor cells. Consequently, strategies aimed at targeting MDSCs have gained prominence in research, presenting new avenues for cancer immunotherapy [261].

A glioma’s blood supply, which translates into aggression, infiltration, and metabolism, is based on two pillars: vessel co-option and angiogenesis [262]. VEGF signaling constitutes a crucial mechanism underpinning tumor angiogenesis, and the attenuation of this pathway has demonstrated the potential to impede tumor proliferation in various preclinical tumor models [263]. Vessel co-option, delineated as the migration of neoplastic cells towards and alongside pre-existing vascular structures, emerges as an alternative strategy to angiogenesis for neoplasms to acquire the necessary nutrients. Upon the ex vivo cultivation of GBM stem cells (GSCs) under endothelium-conducive conditions, these cells exhibited the expression of canonical endothelial markers, including CD31, von Willebrand factor (vWF), and Tie-2. Endothelial cells facilitate the manifestation of the GSC phenotype within the perivascular niche, primarily through direct cellular interactions. This facilitation is achieved by the activation of the Notch signaling pathway in GSCs, attributed to the endothelial expression of Notch ligands and the subsequent release of nitric oxide [264,265,266,267,268]. The majority of patients with GBM inevitably encounter tumor recurrence, predominantly manifesting within 1 to 2 cm of the initial tumor margin. This phenomenon is attributed, in part, to the process of vessel co-option [269,270]. Several molecular pathways have been identified to contribute to this: 1. the EGFRvIII Pathway, 2. the CXCR4/SDF1α Pathway, 3. the Ang-2 Pathway, 4. the IRE-1α Pathway, 5. the CDC42 Pathway, 6. the Olig2/Wnt7a Pathway, 7. the MDGI/FABP3 Pathway, 8. the IL-8 Pathway, 9. the EphrinB2 Pathway, and 10. the Bradykinin Pathway [271,272,273,274,275,276,277,278,279,280,281,282,283,284]. Bradykinin, an endogenous constituent of the cerebral milieu, exhibits augmented concentrations concurrent with tumor progression. Furthermore, GBM cells, particularly those engaging in vessel co-option, demonstrate a pronounced upregulation of the bradykinin receptor-2 (B2R) [285]. Additionally, CXCR4 has been identified as a pivotal chemotactic cue within neoplastic contexts, with a noted overexpression in infiltration GBM cell populations [286]. Moreover, according to the findings of Voutouri et al., who utilized a glioma model to determine the tumor’s response to antiangiogenic therapy, it is better to administer an alternative treatment of antiangiogenic and anti-cooption drugs, not simultaneously [262].

The expanding understanding of tumor immunology has spurred the development of innovative therapeutic modalities and immunotherapies, such as immune checkpoint inhibition (ICI), which has transformed the treatment landscape for a variety of cancers. Novel immunotherapeutic strategies, including chimeric antigen receptor (CAR) T cells, vaccine-based treatments, and oncolytic virus therapies, are undergoing intensive research. However, for patients with GBM, these new therapies have yet to demonstrate a significant survival advantage over the established standard of care [287].

Conversely, CAR-T therapy involves the ex vivo genetic modification of a patient’s T cells to express receptors that target specific tumor antigens. While CAR-T therapy has yielded promising results in preclinical trials, it has not yet proven effective in clinical settings for GBM [288,289,290]. Multifunctional CAR T cells, co-expressing IL12 and IFNα2 in addition to the CAR, exhibited enhanced antiglioma activity both in vitro and in vivo in three orthotopic immunocompetent mouse glioma models, without any observable toxicity. The synergistic action of IL12 and IFNα2 with the CAR was found to foster a proinflammatory TME and mitigate T-cell exhaustion, as evidenced by ex vivo immune phenotyping, cytokine profiling, and RNA sequencing. These multifunctional NKG2D CAR T cells demonstrated potent antiglioma activity, surpassing the efficacy of T cells expressing either the CAR or cytokines alone [291].

Additionally, another study explored the augmentation of CAR-T cell efficacy through the use of an oncolytic adenovirus (oAds) armed with the chemokine CXCL11. This strategy aimed to enhance CAR-T cell infiltration and reprogram the immunosuppressive TME, thereby boosting therapeutic effectiveness. In both immunodeficient and immunocompetent orthotopic GBM mouse models, B7H3-targeted CAR-T cells alone were insufficient to inhibit GBM growth. However, the intratumoral administration of CXCL11-armed oAd in conjunction with CAR-T cells resulted in a durable antitumor response. The oAd-CXCL11 also exhibited a strong antitumor effect and reprogrammed the immunosuppressive TME in GL261 GBM models, characterized by increased infiltration of CD8+ T lymphocytes, NK cells, and M1-polarized macrophages and reduced populations of MDSCs, Tregs, and M2-polarized macrophages. Importantly, the antitumor effect of oAd-CXCL11 was found to be CD8+ T-cell-dependent [292]. Lp2-CAR-T cells were also effective against patient-derived GSCs, indicating potential clinical applicability against GBM. The systemic administration of Lp2-CAR-T cells curbed the growth of a subcutaneous glioma xenograft model in immunodeficient mice. The combined use of Lp2-CAR-T cells and the oncolytic virus G47Δ, a third-generation recombinant herpes simplex virus (HSV)-1, further suppressed tumor growth and improved survival, suggesting the promise of this combination therapy for GBM treatment [293].

Furthermore, αvβ3 CAR-T cells demonstrated efficient antigen-specific tumor cell eradication in cytotoxicity assays and in vivo models with orthotopically and stereotactically implanted DIPG and GBM tumors in the brains of NOD scid gamma (NSG) mice. The tumor responses were swift and pronounced, marked by systemic CAR-T cell proliferation, enduring persistence, and long-term survival. Notably, TCF-1+αvβ3 CAR-T cells were detectable post-tumor clearance, highlighting their potential for self-renewal and sustained presence [294].

CAFs represent an important type of cell inside LGGs. A study by Dong et al. showcased that, by methylating some expressed genes of CAFs, the sensitivities to immune checkpoint blockade treatment varied when chemotherapeutic agents were administered [183]. Moreover, the general assumption about the immunological standpoint of CAFs was that of an immunosuppressive cell; however, this statement is debated, with recent evidence supporting the contrary [295]. Therapeutic strategies are based on the classic assumption that CAFs act as immunosuppressive agents [296,297]. Subsequently, a study performed in 2019 aimed to demonstrate that, through the utilization of Angiotensin receptor blockers, myofibroblasts that developed from CAFs were able to be reversed back to the immunosupportive state [298]. Moreover, CAFs expression inside the solid tumor can be utilized as a prognostic tool for the overall evolution and treatment response of the cancer, according to a 2021 analysis [299].

Drugs often used in the field of psychiatry have demonstrated anti-tumoral effects, and represent a viable treatment option in GBM, not only because of their high BBB permeability, but also because of their peculiar interactions with GBM. The administration of chlorpromazine led to a reduction in the expression of cell-cycle-associated proteins, including cyclin D1, cyclin A, and cyclin B1, concurrently with an elevation in the expression of tumor suppressor genes such as early growth response (*EGR*)-1 and *P21* [300,301,302,303]. This regulatory shift precipitated cell cycle arrest. Furthermore, evidence supports that chlorpromazine induces autophagic cell death in GBM cells derived from patients. This phenomenon is attributed to the involvement of reactive oxidative stress (ROS), endoplasmatic reticulum (ER) stress, and the unfolded protein response in this process [302,304].

Trifluoperazine is posited to exert its effects through modulation of the inositol triphosphate receptor (IP3R), facilitating the release of Ca^2+^ from the ER. This action results in an elevated intracellular concentration of Ca^2+^, disrupting calcium homeostasis and inhibiting glioma cell invasion and proliferation [305]. Additionally, trifluoperazine interferes with the interaction between *IDH* and calmodulin, thereby impeding the survival and migration of GBM cells harboring wildtype *IDH1* [306,307].

Tumor Treating Fields (TTFields) are electric fields that interfere with key cellular mechanisms necessary for the survival and growth of cancer cells, ultimately inducing cell death. TTFields therapy has been authorized for the treatment of newly diagnosed GBM in conjunction with maintenance temozolomide. Recent evidence has shown the efficacy of TMZ when combined with lomustine (CCNU) in patients with O6-methylguanine DNA methyltransferase (MGMT) promoter methylation, findings which reinforce the clinical advantage of using TTFields in combination with both temozolomide and lomustine [308].

Another study investigated the efficacy of adding Autologous tumor lysate-loaded DC vaccination (DCVax-L) to standard-of-care temozolomide in treating GBM. Patients with newly diagnosed glioblastoma (nGBM) in the active treatment group received DCVax-L plus SOC temozolomide, while external control patients with nGBM were administered SOC temozolomide and a placebo. Recurrent glioblastoma (rGBM) external controls received approved rGBM therapies. The primary and secondary outcomes focused on comparing the overall survival (OS) in both the nGBM and rGBM patient groups with matched external control populations from other formal randomized clinical trials. The results indicated that the addition of DCVax-L to SOC significantly prolonged survival in patients with both nGBM and rGBM, compared to matched external controls who received only SOC [309]. Currently, another clinical trial of DCVax-L is ongoing (NCT00045968).

## 11. Comparative Studies: Gliomas vs. Other Tumor Entities

Comparative oncology clinical trials, particularly those conducted in pet dogs, are increasingly integral to cancer research and the development of new anticancer drugs. These trials enable the evaluation of novel agents and therapy combinations within a veterinary clinical framework conducive to repeated biological sample collections and the study of dosage, timing, and their pharmacokinetic/pharmacodynamic outcomes [310].

This interdisciplinary approach is dedicated to the betterment of both human and veterinary medicine, with significant strides evident in the comparative analysis of canine and human tumors. Canine cancers occurring naturally offer a valuable model that can enhance the understanding, diagnosis, and treatment of human cancers [311,312].

However, the progress in treating HGG like GBM has been minimal. The formidable challenges include the highly proliferative and invasive nature of GBM, which hinders complete surgical removal and diminishes the effectiveness of traditional therapies, and the extensive intra- and inter-tumoral heterogeneity, which complicates the identification of consistent therapeutic targets [313].

Malignant gliomas are characterized by their uncontrolled proliferation, aggressive infiltration into adjacent brain tissue, robust neovascularization, necrotic regions, and a notable resistance to programmed cell death. Their propensity to diffusely infiltrate the brain parenchyma, intertwining with healthy cells, renders complete surgical removal infeasible [314]. Currently, the marginally effective approach to extending life expectancy in glioma patients involves surgical resection, complemented by radiation and chemotherapy using temozolomide. Despite these interventions, tumor recurrence is common, typically within 6.9 months, leading to a median survival time of only 12 to 15 months post-diagnosis [315]. Moreover, associating temozolomide with alfa interferon increases the overall survival in patients [316].

Dexamethasone, a potent glucocorticoid, has showed great antitumoral properties in different cell lines specific to cancers like colon or breast cancer [317,318]. In the case of GBM, however, the use of dexamethasone is debated. Brain edema is one of the factors that renders neurological disability in patients with brain tumors, for which dexamethasone was commonly utilized in this kind of situation in standard treatment [319]. It appears that dexamethasone is a therapeutic agent that might be detrimental in GBM treatment, decreasing T lymphocyte and natural killer cell numbers. A study conducted by Iorgulescu et al. showed that dexamethasone administration was correlated with a low survival rate in patients with GBM [320]. Moreover, dexamethasone was shown to increase malignancy in GBM cell lines due to an overall increase in glucose levels [321]. On the other hand, other studies testify in favor of dexamethasone’s use in GBM therapies, studies that showcase the benefic effects of inhibiting the GBM cell infiltration of subsequent tissues [322]. Even though dexamethasone is used with great results in numerous therapies, we must keep in mind that still unknown interactions may exist between cancerous cells and dexamethasone, and, as a result, further research must be performed regarding dexamethasone–GBM interactions in order to reach a consensus.

Targeting immune checkpoints has been a great strategy in the treatment of cancers. One immune checkpoint of potential interest in cancers is represented by PD-1 and its co-inhibitory factor programmed cell death protein ligand 1 (PD-L1) and PD-L2 [323,324]. PD-1/PD-L1 is an axis that is present in numerous types of cancers and utilizes different signaling pathways to elude the body’s immune response. Exploiting these signaling pathways has showed promising results in cancer therapy: for example, PD-L1 increases PI3K/AKT expression in colorectal cancer cells [325]; as a result, PD-L1 targeted therapy has been developed in the form of Nivolumab [326,327,328]. Moreover, a blockade of the MAPK signaling cascade effectively thwarted the upregulation of CD274 mRNA and PD-L1 protein and membrane expression, instigated by EGFR and IFN-γ in lung adenocarcinoma cells [329]. Nivolumab and pembrolizumab, through their targeted inhibition of the PD1 receptor and subsequent blockade of its interaction with PDL1 and PDL2, have received authorization for the treatment of advanced non-small lung cancer [330,331]. Additionally, the employment of selective WNT pathway modulators, either inhibitors or activators, to decrease or augment PD-L1 expression, respectively, is used in the treatment of triple-negative breast cancer [332]. Regarding GBM, it is interesting to note the antithetic results of nivolumab treatment: nivolumab alone or in combination with radiotherapy or ipilimumab has not been as successful in the treatment of gliomas, according to numerous clinical trials conducted recently [333,334,335]. However, on the other hand, other clinical studies and case reports have yielded a greater survivability of patients treated with pembrolizumab [336] or with a combination of nivolumab and pembrolizumab [337].

## 12. Future Directions in Glioma Research and Conclusions

The integration of genomics and molecular profiling into personalized treatment strategies for gliomas has been a significant development. A study identified a prognostically relevant signature comprising 17 lncRNAs associated with genomic instability, offering potential therapeutic implications for GBM. These lncRNAs (including LINC01579, AL022344.1, and CRNDE, among others) were linked to different survival outcomes, with the lower-risk group exhibiting better survival rates. This lncRNA signature also stood out as an independent risk factor across various clinical stratifications, with most patients in the lower-risk group exhibiting mutations in *IDH1*. Furthermore, these lncRNAs were expressed at higher levels in GBM cell lines compared to normal cells, indicating their potential role in tumor pathology [338].

In the realm of immunotherapy, checkpoint inhibitors and chimeric antigen receptor (CAR) T cell therapies have generated optimism through their preclinical successes. Clinical trials continue to progress, aiming to identify optimal treatment approaches [339,340,341,342].

Additionally, the advent of artificial intelligence (AI) in medicine, particularly its application in analyzing complex, high-dimensional images of brain tumors, presents new frontiers in understanding and treating brain tumors. AI’s capacity to enhance the accuracy of tumor genotyping, delineate tumor volumes precisely, and improve clinical outcome predictions exemplifies the convergence of AI with precision medicine, setting the stage for transformative changes in healthcare [343,344].

In concluding this comprehensive exploration of gliomas, we traversed the complex landscape of these relentless brain tumors, illuminating the various facets that constitute our current understanding. Proteomics, epigenetics, and genetics play key roles in gliomas, with particular focus on the critical role of glial cells and the significance of cellular communication within the tumor milieu. We proceeded to unravel the genetic factors contributing to these malignancies, and how advances in proteomics and metabolomics have elucidated the modifications in protein expression and metabolic shifts, opening up avenues for therapeutic interventions. The discourse also encompassed the influence of the TME in gliomas and the promise held by targeted therapies and comparative oncology.

Moving forward, the future of glioma research will pivot towards evaluating emerging breakthroughs and innovative strategies on the horizon. The upcoming focus will be on what the medical community must achieve to mitigate the devastating impact of gliomas, with the ultimate goal of eradicating this formidable disease. It is important to keep in mind that no single mechanism is responsible for the development of these dreaded neoplasms. A plethora of intricate factors, co-stimulating mechanisms, and much more hold the key to understanding such a complex pathology. In our search for an answer for this disease, we must always keep in mind the bigger picture while continuing our research endeavors.

## Data Availability

Not applicable.

## Abbreviations

WHO	World Health Organization
CNS	Central nervous system
HGG	high-grade gliomas
LGGs	Low-Grade Gliomas
MRI	Magnetic resonance imaging
GBM	Glioblastom
*IDH1*	Isocitrate dehydrogenase 1
IHC	Immunohistochemistry
UPS	Ubiquitin proteasome system
AS	Astrocytoma
ODG	Oligodendroglioma
HDAC1	Histone deacetylase 1
NSCs	Neural stem cells
GSCs	Glioma stem cells
SVZ	Subventricular zone
OPCs	Oligodendrocyte progenitor cells
PUFAs	Polyunsaturated fatty acids
NAC	N-acetylcysteine
PE-PUFAs	Phosphatidylethanolamine PUFA
BBB	Blood-brain barrier
FasL	Fas ligand
TAAs	Tumor-associated antigens
MDSCs	Myeloid-Derived Suppressor Cells
CSF-1	Colony Stimulating Factor-1
Arg1+	Arginase 1 positive
*AEG1*	Astrocyte elevated gene-1
TCGA	The Cancer Genome Atlas
GTEx	Genotype-Tissue Expression
CGGA	Chinese Glioma Genome Atlas
MIF	Macrophage migration inhibitory factor
PD-1	Programmed cell death protein 1
Tregs	Regulatory T cells
Tfr	T follicular regulatory
CTLA-4	Cytotoxic T-Lymphocyte Associated protein 4
PFG	Permeability Factor G
NK	Natural Killer
TGF-β	Transforming Growth Factor Beta
NKG2D	Natural-killer group 2, member D
IFN-γ	Interferon Gamma
CAR-T	Chimeric Antigen Receptor T
*EGFRvIII*	EGFR variant III
IL-13Rα	Interleukin-13 Receptor Alpha 2
TME	Tumor microenviroment
Treg	Regulatory T cell
TIM-3	T-cell immunoglobulin domain and mucin domain-3
DCs	Dendritic cells
DIPG	Diffuse Intrinsic Pontine Glioma
SRS	Stereotactic Radiosurgery
CSCs	Cancer stem cells
TAMs	Tumor-associated macrophages
EVs	Extracellular vesicles
CX-43	Connexin-43
JNK	C-Jun N-terminal kinase
Grnd	Grindelwald
*Egr*	Eiger
BDNF	Brain-derived neurotrophic factor
GPER	G protein-coupled estrogen receptor
GICs	Glioma-initiating cells
AQPs	Aquaporins
*EGFR*	Epidermal growth factor receptor
ABAT	Aminobutyrate aminotransferase
*PDGFRA*	Platelet-derived growth factor receptor alpha
RNA	Ribonucleic acid
TFs	Transcription factors
lncRNA	Long non-coding RNA
DNA	Deoxyribonucleic acid
PTMS	Posttranslational modifications
SENP1	SUMO-specific protease
miRNA	MicroRNA
DII1-4	Delta-like family
PKB	Protein kinase B
PI3K	PI3 kinase
ACSS2	Acetyl-CoA synthetase 2
SREBP	Sterol regulatory element-binding protein-1
EOR	Extent of resection
LOS	Length of hospital day
CSR	Complete surgical resection
IORT	Intraoperative radiation therapy
IoMRI	Intraoperative MRI
ECM	Extracellular matrix
TREM2	Triggering receptor expressed on myeloid cells 2
CAFs	Cancer-associated fibroblasts
FN1	Fibronectin
MDSCs	Myeloid-Derived Suppressor Cells
VEGF	Vascular endothelial growth factor
vWF	Von Willebrand factor
B2R	Bradykinin receptor-2
ICI	Immune checkpoint inhibition
CAR	Chimeric antigen receptor
oAds	Oncolytic adenovirus
DIPG	Diffuse Intrinsic Pontine Glioma
NSG	NOD scid gamma
*EGR*	Early growth response
*ROS*	Reactive oxidative stress
ER	Endoplasmic reticulum
IP3R	Inositol triphosphate receptor
TTFields	Tumor Treating Fields
CCNU	Lomustine
*MGMT*	Methyltransferase
DCVax-L	Dendritic cell vaccination
nGBM	Newly diagnosed glioblastoma
rGBM	Recurrent glioblastoma
OS	Overall survival
PD-1	Programmed cell death protein
PD-L1	Programmed cell death protein ligand 1
ncRNAs	Non-coding RNAs
AI	Artificial intelligence
